# Creating a social movement to engage communities in physical activity: A mixed methods study of motivations to engagement

**DOI:** 10.1371/journal.pone.0263414

**Published:** 2022-02-10

**Authors:** Marc Harris, Diane Crone, Samantha Hughes, William Bird

**Affiliations:** 1 Cardiff Metropolitan University, Cardiff, England; 2 Intelligent Health, Waltham Cross, England; 3 University of Gloucestershire, Cheltenham, England; La Inmaculada Teacher Training Centre (University of Granada), SPAIN

## Abstract

Throughout the world social isolation and loneliness are common and both have several adverse impacts on health and wellbeing. We are designed to live in close-knit communities and we thrive in close co-operation, however, modern life isolates us from others. To reduce the burden of loneliness and social isolation we need to find strategies to reconnect people to each other, their place and provide a common purpose. Social movements aim to create healthier communities by connecting people to each other and giving people a common purpose. Interventions which create a social movement appear to be effective at engaging substantial portions of a community, however, it remains unclear why individuals are attracted to these initiatives, and if such reasons differ by sociodemographic characteristics. This study combined qualitative and quantitative methods to understand what motivated (different) people to take part in a social movement based intervention. This study suggests that it is not one but a combination of reasons people engage in interventions of this nature. This diversity needs to be acknowledged when promoting and communicating these interventions to potential participants to maximise engagement. Promoting an end reward or health/fitness may not be the most effective way to promote interventions to a large proportion of people. Instead, communications should be centred around what people value (i.e., being with their friends, doing what they enjoy and are good at).

## Introduction

Throughout the world, social isolation (an objective lack of interactions with others or a wider community) and loneliness (a subjective feeling of the absence of a companion or social network) are common [1]. Surveys in Europe, the USA and China estimate that between 5% and 43% of elderly populations suffer from loneliness [1–6]. Loneliness may be more common in older groups however, it also affects people across society and every individual will experience loneliness at some point in their lives to a certain degree [7,8]. Research has shown that only 22% of individuals never feel lonely and one in ten people often feel lonely.^7^ Prior to the SARS-CoV-​2 (Covid-19) pandemic in 2020, the prevalence of loneliness and social isolation led some to describe it as the ‘behavioural epidemic’ [9,10]. Although since this pandemic the situation has worsened. A report published by University College London and What Works Wellbeing suggests young adults (aged 18–30), people with low household income and those living alone were at heightened risk of loneliness during the pandemic [11].

Loneliness and social isolation have several adverse effects on health and wellbeing. They have been associated with poorer sleep quality, increased depression symptomology, impaired functioning, reduced quality of life, suicide, dementia, increased risk of coronary artery disease, and all-cause mortality [12–17]. A causal pathway for the adverse health impacts of loneliness and social isolation has been proposed which implicates increased cortisol due to a stress response [18]. Bosch and Bird explain that everyday stressors lead to chronic stress and poorer health behaviours, which in turn lead to chronic inflammation and chronic health conditions [19]. Evolution tells us that we were designed to live in close-knit communities and sociology informs us that we thrive in close co-operation with others, however, our modern way of life isolates us from others [7]. This isolation from others can induce a behavioural response (such as drug or alcohol use, smoking, physical inactivity, and poorer dietary choices) [1,20]. Bosch and Bird argue that people have become disconnected from each other and their environment and as a result feel a lack of purpose [19]. We need to find strategies and interventions to reconnect people to each other, their place, and provide a purpose to reduce the negative impact of social isolation and loneliness [19].

Social prescribing and social movements represent two approaches which aim to reduce chronic inflammation by connecting people to each other, giving people a common purpose, and delivering place-based care [19]. Social prescribing typically uses a ‘link worker’ who connects people with existing community groups with structured activities (such as health walks, organised running events in parks, exercise classes, walking sports, horticultural activities, etc) in addition to providing support for other aspects of their lives (for instance, navigating benefits schemes, getting job training, assisting with the writing of a curriculum vitae, etc) [21]. Social movements are not new and devolve delivery and control to the community. Instead of a link worker referring a patient to an organised activity, the social movement simply connects an individual to a self-created unstructured activity. One example of an evidence-based intervention that uses the social movement model is ‘Beat the Street’, a mass participation intervention that aims to get people more active, increase social cohesion and connect people to their local neighbourhood. It combines gamification technology and behavioural psychology to engage communities. Between 2015 and 2019, over 1 million people and 2000 schools participated in Beat the Street and on average, 13% of each community targeted engaged with the program [22]. Recent research suggests this approach may improve mental wellbeing, active travel, and physical activity in the short-term and up to 2 years later [23–27]. What currently remains unclear is why individuals are attracted to this type of programme, and if such reasons differ by sociodemographic characteristics. Further, little is known about the relationship between motivations to participation and subsequent behaviour change.

## Materials and methods

### Intervention

Beat the street is a gamification-based intervention developed and delivered by a UK based health-technology organisation, Intelligent Health Ltd. The game uses Radio Frequency Identification (RFID) scanners called ‘Beat Boxes’ which are situated at half-mile intervals throughout a community. Each time 2 boxes are touched with a RFID card or fob within 1 hour a player receives 10 points for themselves and a chosen team. The game incentivises participation by allowing teams to win prizes (such as vouchers for sports equipment in schools), individuals to win random spot prizes by tapping a Beat Box (i.e., a £25 voucher), and for a community to collectively raise money for a local charity by achieving a specific target (i.e., £500 for travelling 50,000 miles collectively). Players monitor their own, their teams and the whole communities progress via an online portal and several leader boards. The challenge runs continuously for 6 weeks. This study was conducted with participants taking part across seven separate programmes delivered in towns and cities throughout England. These included Barnsley, Blaby, East Northamptonshire, Gloucester, Leicester, Salisbury, and West Sussex. As such, the areas and participants taking part in this study were both geographically and demographically diverse.

### Participants and procedure

The methodological approach adopted by this study closely reflected that of Hughes and colleagues [28]. A sequential mixed methods design was used, whereby quantitative analyses were used to develop a greater understanding of qualitative data obtained through an open question [29]. To elaborate, when registering to take part in the game, players were asked to complete a self-report questionnaire which included a range of demographic questions (i.e., age, gender, ethnicity and disability), a validated physical activity questionnaire [30] and an open question “What motivated you to take part in Beat the Street?”. During the registration process, participants provided written informed consent. For those aged 13 and under, informed consent was provided by a parent or guardian. Following the intervention, players were invited to complete the self-report physical activity questionnaire (Short-Active Lives Survey) again, which provided a measure of behaviour change achieved through the intervention. Across the 7 game areas that formed this study, 1,526 players completed the sociodemographic questionnaire, provided data on their physical activity at baseline and follow-up, and provided qualitative feedback on why they took part in the intervention. Of these, 60% were aged between 30–49, 72% were female, 92% were of a white ethnic background, 2% had a disability. During registration (prior to the game), 20% were inactive and 67% were meeting the World Health Organisation target of 150+ minutes of moderate intensity activity per week. Following the game, the proportion reporting being inactive decreased to 13% (P<0.001) and the proportion undertaking 150+ minutes of moderate intensity activity per week increased to 73% (P<0.001). A more detailed overview of sample characteristics is provided in Table 1.

**Table 1:** Sample characteristics.

Indicator	Respondents
**Age**	
11 and under	12%
12 to 18	1%
19 to 29	6%
30s	30%
40s	30%
50s	10%
60s	7%
70s	2%
80s	0.1%
Prefer not to say	1%
**Gender**	
Female	72%
**Ethnicity**	
White	92%
**Disability**	
Yes	2%
**Multiple Deprivation (Quintile)**	
High	14%
Mid-high	17%
Mid	19%
Mid-low	24%
Low	25%
**Baseline Activity Status**	
Inactive (0-29mins per week)	20%
Fairly active (30-149mins per week)	13%
Active (150+mins per week)	67%
**Post-intervention Activity Status**	
Inactive (0-29mins per week)	13%
Fairly active (30-149mins per week)	14%
Active (150+mins per week)	73%
**Location**	
Barnsley	16%
Blaby	18%
East Northamptonshire	18%
Gloucester	6%
Leicester	8%
Salisbury	15%
West Sussex	20%

### Method of analysis

Firstly, qualitative data obtained through the open question “What motivated you to take part in Beat the Street?” were analysed through thematic analysis [31,32]. There were six stages to the analysis which included (i) the third author reading and re-reading open qualitative feedback and creating notes of initial themes of interest throughout the process; (ii) the third author generated initial codes by systematically reading the transcripts twice, line-by-line, and noting interesting features within the data; (iii) the third author collating codes into themes with quotes attached to each theme; (iv) all authors then collectively reviewed and refined each theme and supporting evidence; (v) the fourth author then reviewed the themes and defined them and identified an agreed theme title (name); (vi) the final stage involved all authors deciding on the selected quotations which best explained each theme.

Following the thematic analysis, the first author linked each quotation and the theme which best represented their motivation to each participant, alongside their demographic characteristics (Table 1). At this stage, data were analysed quantitatively, whereby the proportion of participants reporting each motivation (or theme) was compared between different demographic characteristics. The independent variables were each of the demographics listed above in Table 1 (gender, age, disability status, ethnicity, index of multiple deprivation, baseline activity level, and behaviour change). The dependant variable was each participants motivation for taking part in the intervention (school initiative, health and fitness promotion, incentives, or social/familial activity). Data were analysed using a series of Chi-Squared tests.

## Results

### Qualitative component of the study

Four themes (motivations) were identified through thematic analysis, including: (1) A school initiative, (2) Health and wellbeing promotion, (3) Incentives, and (4) Social/Familial activity. Within each theme were dimensions which help to provide a nuanced understanding of the primary reasons for engaging with the programme. Each of these themes is explained below and are presented, with supporting quotations, in Table 2 to enable the voice of the participants to be heard within the findings.

**Table 2:** Motivations to engagement with Beat the Street.

Superordinate Theme	Subordinate Themes	Evidence
1. **A school initiative**	**School promotion**	“My child came home with the pack from school. I had read about it in the paper but I probably wouldn’t have got round to picking up a card”–Female, aged between 40–49 “I was given my fob at school and strongly liked the idea. I really feel confident for my school and hope they will win.”–Female, aged under 12.
	**Supporting a school initiative**	“To get my child more active and earn points for her school”–Female, Aged between 30–39. “I want to help motivate our school children by showing that healthy activity is something everyone can and should participate in.”–Male, Aged between 30–39. “To help the school and be a good role model for children”–Female, aged between 60–69.
2. **Health and fitness promotion**	**Facilitate health and fitness**	“Help me to lose weight whilst also getting more fresh air”–Female, Aged between 30–39. “Thought it was a good idea to help to keep fit”–Male, Aged between 70–79.
	**To be (more) physically active**	“I enjoy running occasionally and would like to run more regularly”–Male, Aged between 40–49. “I like to walk and could do with increasing how much I do so this is a good incentive.”–Female, Aged between 50–59.
3. **Incentives**	**Competing with others and oneself**	“It gives you a sense of purpose and I like a challenge”–Female, aged between 70–79. “The challenge to visit as many (boxes) as possible”–Female, Aged between 40–49. “Getting out of the office & competition, to out-do fellow staff”–Female, Aged between 50–59.
	**To gain rewards**	“To help raise money for the school and charities”–Female, Aged between 40–49. “To see how many points I can clock up”–Female, Aged under 12. “My children want to earn points!”–Female, Aged between 30–39.
4. **Social/Familial activity**	**A generational family activity**	“Grandson asked me too—but it is a brilliant idea anyway!”–Male, Aged between 70–79. “My partner. We both want to get out and walk more. It also give us a chance to take our new-born out as well.”–Male, Aged between 30–39.
	**Supporting children**	“My little girl and her friends gave me the motivation to take part in this activity.”–Female, Aged between 30–39. “My child is taking part & wants us to help out with points”–Female, Aged between 40–49. “To encourage my visually impaired daughter to get more exercise.”–Female, Aged between 40–49.
	**Taking part with other people**	“Get out on my bike more and help my little brothers team”–Male, Aged between 12–18. “Joining in on something that the whole community can get involved in and to try & be more active”–Female, Aged between 60–69. “Challenge for the adults I support with Aldingbourne Trust.”–Female, Aged between 50–59.

### 1. A school initiative

The Beat the Street intervention was promoted heavily through schools and involved schools competing against each other to win prizes. Thus, motivation to support the initiative due to a connection with the school was a key reason to take part. There were a number of dimensions to this theme; firstly, parents and children felt obliged to take part because they were sent communication in the form of letters, emails, and social media promotion from their (child’s) school asking them to take part. Secondly, children and parents wanted to help their school beat other schools in their community and to win prizes for their school. Further, adults felt the scheme could help set a good example to the school children by taking part in an activity which encourages health promoting behaviours.

### 2. Health and fitness promotion

Other players were motivated by the health and wellbeing benefits they could gain from taking part in the intervention. This motivation ranged from getting outside and having fresh air, losing weight, getting fit, walking more, and encouraging running more regularly. In this sense, players were attracted to the game because it generated a sense of purpose for them, and for some this involved being able to track the amount of exercise they were undertaking via the game itself.

### 3. Incentives

This initiative was gamified through points achieved by tapping cards at the various Beat Boxes and accumulating points which were then displayed on a leader board. These points were then converted into prizes for the highest scoring teams at the end of the game. As such, external incentives (i.e., points and prizes) were a key motivator for some players. There were two elements to this theme. For some participants, the incentive was the challenge itself, and competing with others (such as friends and colleagues), or simply trying to visit every Beat Box within the game. For others, the challenge was to gain as many points as possible for themselves and their children, and in doing so helping to raise money for a local charity.

### 4. Social/Familial activity

The final theme concerned the social nature of the intervention. Many of the players were engaged with the intervention as it provided an activity that people with differing demographic profiles and physical and mental abilities could take part in. There were three aspects to this motivation. Firstly, the game provided an intergenerational family activity. For example, grandparents could take part with their children and grandchildren, or a parent or parents could take part with a child(ren) of any age (for example, a baby in a pram or a child on their scooter). Secondly, families were motivated to support their child(ren), where they wanted to help their child(ren) succeed in the game or to support them to be more active. Thirdly, at a broader community-wide level, participants wanted to take part with the rest of their town or city. This motivation ranged from helping a sibling or distant relative gain points for their team, to supporting people with learning disabilities be more engaged with a community initiative, for example.

### Quantitative component of the study

Adopting the sequential mixed methods approach to this study, the next stage sought to understand the most or least prominent motivations for participating in the intervention, and identify if the importance of different motivations varied between participant demographics. Participants were segmented by gender, age, ethnicity, whether or not they had a disability, index of multiple deprivation (based on their postcode of residence), pre-intervention level of physical activity, and change in physical activity following the scheme (i.e., if levels had increased, stayed the same, or decreased) (See Table 3 for a segmentation of motivations by participant demographics).

**Table 3:** Motivations between participant demographics.

Participant Demographics (N = 1526)		School Initiative	Health and Wellbeing Promotion	Incentives	Social/Familial Activity
Overall	Overall	33%	13%	10%	44%
Gender	Female	34%	13%	10%	43%
	Male	29%	15%	10%	46%
Age	Adult	30%	14%	7%	49%
	Child	51%	8%	27%	14%
Disability	Yes	25%	18%	8%	48%
	No	33%	13%	10%	44%
Ethnicity	White (Welsh/English/Scottish/Northern Irish/British or any other White ethnic group)	33%	13%	10%	44%
	Black, Asian and other culturally diverse communities	28%	23%	11%	39%
Index of Multiple Deprivation	First quintile (Highest)	30%	12%	5%	53%
	Second quintile	31%	14%	8%	47%
	Third quintile	29%	9%	12%	51%
	Fourth quintile	37%	13%	9%	40%
	Fifth quintile (Lowest)	32%	16%	13%	39%
Baseline Activity Level	Inactive	36%	11%	7%	46%
	Fairly active	36%	12%	7%	44%
	Active	31%	14%	11%	43%
Behaviour Change	Inactive (<30mins) to Active (>29mins)	34%	14%	5%	47%
	<150mins to >150mins	37%	11%	10%	42%
	No Change	32%	14%	11%	43%
	Decrease in Activity	29%	13%	13%	45%

Overall (regardless of any within group differences), school-based motivations and social/familial motivations were the most common reasons for taking part in the game, reported by 33% and 44% of players, respectively. Only a small proportion (10%) of players were motivated by incentives (i.e., points and prizes) and only 13% were motivated by health and wellbeing benefits. These proportions did not differ significantly by gender, (χ^2^ = 4.40, df = 3, p > .05), disability status (χ^2^ = 2.56, df = 3, p > .05), ethnic background, (χ^2^ = 4.42, df = 3, p > .05), pre-intervention level of physical activity (χ^2^ = 11.69, df = 6, p > .05) or behaviour change (χ^2^ = 12.32, df = 9, p > .05). There were, however, certain demographics where these proportions differed significantly. These were age (χ^2^ = 141.44, df = 3, p < .05) where children were motivated more by incentives and less by social/familial reasons, and index of multiple deprivation (χ^2^ = 31.04, df = 12, p < .01), where those living in less affluent areas were motivated more by social/familial reasons and less by incentives.

## Discussion

The findings presented in this study provide novel insight into the factors that motivate people into community level physical activity initiatives. At a broad level, this research suggests that initiatives which seek to engage communities in physical activity should focus on encouraging social participation. That is, enabling different groups (i.e., older versus younger), family units, and colleagues, to take part in an activity together and with the same rules, rituals, or expectations. Further, physical activity programmes could reach substantial portions of a given community by utilising schools as a fundamental community asset. Many people living in a community are often closely connected to a local school, and schools can be an effective method of communication to many people in the community. As such, schools are uniquely placed to publicise and stimulate interest in community wide initiatives, whilst also being mindful that residents who do not have children nor grandchildren living nearby may be excluded from some promotion. The findings also imply that whilst incentives (i.e., winning prizes) and achieving health benefits, are important for some people (23% of participants studied here), a substantial proportion of residents may not be enticed into initiatives for these reasons. Thus, the ‘health by stealth’ approach, whereby people achieve health benefits more subconsciously through activities where health is scarcely mentioned, may be more effective at engaging large proportions of a community into interventions.

At a microlevel, these assumptions will shift. This research found the importance of each motivation remained stable when data were segmented by different demographic characteristics (i.e., gender, disability, or pre-existing levels of physical activity). However, certain groups were motivated to a greater degree by specific reasons. Children, for instance, were motivated more by incentives than players overall, and were less motivated by social or family reasons, and those who lived is less affluent areas were motivated more by social and family reasons and less by incentives (i.e., winning prizes) than players overall. This suggests initiatives need to be tailored to meet the needs and expectations of different groups within a community.

### Literature contributions

This study suggests that it is not one, but a combination of reasons people engage in interventions of this nature. This diversity needs to be acknowledged when marketing and communicating these interventions to potential participants to maximise engagement. Further, the results of this study are somewhat counterintuitive, in the sense that the game itself (i.e., gaining points, moving up the leader board, and winning prizes) motivated just 1 in 10 people. This research is unable to establish what game components were most influential for keeping people engaged throughout, but it does suggest that encouraging school-based connection, and enabling social and familial networks to take part in something together, are most effective at inciting people into mass participation initiatives. Thus, it is argued that to reach substantial portions of a community, interventions (both gamification and non-gamification based) should seek to promote collective social action (a social movement) rather than solely promoting an end reward, such as winning a competition. This study also suggests that promoting health or fitness may not be the most effective way to promote physical activity interventions to a large proportion of people. Instead, ‘stealth marketing’, whereby ‘undercover’ methods of promoting physical activity are used, may be more effective at motivating people to perceive physical activity as fun and rewarding, rather than an additional burden [33–37]. This assumption is supported by a recent qualitative study by Strommer and colleagues who found health was not a motivating factor for adolescents; therefore, interventions designed solely to improve health are unlikely to engage them.^38^ Interventions which are centred around what people value (i.e., being with their friends, doing what they enjoy and are good at) are more likely to be effective [38]. Further, promoting the wider benefits which could be gained by taking part, such as improvements to mental wellbeing or greater social cohesion, could be more effective at enticing people to engage with interventions, rather than solely focusing on physical health or fitness [23]. Supporting this argument, a recent systematic review of physical activity messaging across 123 published studies by Williamson et al. concluded that physical activity messages should be framed positively and highlight short-term outcomes, specifically relating to social and mental health [39].

### Strengths, limitations and future directions

This study combined data from real-world interventions delivered across several diverse areas in one country. In doing so, it has advanced the evidence-base for what works when promoting community wide interventions. Further, this study used a bottom-up approach to understand what motivated individuals into this intervention, rather than providing several, author-led, potential motivations for participants to select. It is worth noting, however, that this approach (mixing qualitative and quantitative methods) can be controversial [40]. Some authors argue that quantitative and qualitative approaches are incompatible because the two approaches are fundamentally different and therefore attempts to merge them would compromise the philosophical foundations of each [41–45]. Whereas others argue that the qualitative/quantitative debate creates a binary distinction that does not hold in practice [40]. For example, counting often involves qualitative judgments, and that numbers often relate to context. Further, qualitative data are sometimes transformed in data analysis into categorical data, and a binary stance overlooks both within-group (e.g., qualitative) and between-group similarities (e.g., qualitative, and quantitative) [40,46]. With this in mind, it was felt that the benefits of mixing both qualitative and quantitative methods, in allowing participants themselves to present what they felt motivated them, rather than what the authors felt motivated them, largely outweighed potential limitations.

This research has identified four primary motivations to engagement in a mass-participation intervention. However, this study is only able to establish what attracted people to sign-up to this intervention, and not what motivated them once they had engaged, nor their continued participation in physical activity beyond the time limitations of the intervention. It is possible that social or school-based motivations could be influential at promoting an intervention to large portions of a community. However, incentives, competition, or health/fitness-based components may be important for keeping people engaged. Further, the relative importance of a given motivation may fluctuate as people move into greater physical activity. For example, a person who lives a predominantly sedentary life may be attracted into a physical activity intervention if it is social in nature, and seldom mentions health and fitness. As they become more active, this person may become motivated more by intrinsic or goal-setting factors (i.e., tracking one’s progress). Future research should seek to understand how motivations develop over-time, as individuals engage in physical activity interventions and become more active.

### Conclusion

This study suggests that it is not one, but a combination of reasons people engage in interventions. This diversity needs to be acknowledged when promoting and communicating these interventions to potential participants to maximise engagement. Further, promoting an end reward, or health/fitness may not be the most effective way to promote interventions to a large proportion of people. Instead, interventions should be centred around what people value (i.e., being with their friends, doing what they enjoy and were good at).

## References

[1] Leigh-Hunt N., Bagguley D., Bash K., Turner V., Turnbull S., Valtorta N., et al. (2017). An overview of systematic reviews on the public health consequences of social isolation and loneliness. Public health, 152, 157–171. doi: 10.1016/j.puhe.2017.07.035 28915435

[2] Office of National Statistics. (2013). Measuring national wellbeing, older people and loneliness. Available from: https://webarchive.nationalarchives.gov.uk/20160106033529/ http://www.ons.gov.uk/ons/rel/wellbeing/measuring-national-well-being/older-people-and-loneliness/art-measuring-national-well-being—older-people-and-loneliness.html [Accessed 22/07/2021].

[3] Dykstra P. A. (2009). Older adult loneliness: myths and realities. European journal of ageing, 6(2), 91. doi: 10.1007/s10433-009-0110-3 19517025 PMC2693783

[4] Sörensen S., & Pinquart M. (2000). Vulnerability and access to resources as predictors of preparation for future care needs in the elderly. Journal of Aging and Health, 12(3), 275–300.

[5] Yang K., & Victor C. R. (2008). The prevalence of and risk factors for loneliness among older people in China. Ageing & Society, 28(3), 305–327.

[6] Perissinotto C. M., Cenzer I. S., & Covinsky K. E. (2012). Loneliness in older persons: a predictor of functional decline and death. Archives of internal medicine, 172(14), 1078–1084. doi: 10.1001/archinternmed.2012.1993 22710744 PMC4383762

[7] Griffin J. (2010). The lonely society. London: Mental Health Foundation.

[8] Fakoya O. A., McCorry N. K., & Donnelly M. (2020). Loneliness and social isolation interventions for older adults: a scoping review of reviews. BMC public health, 20(1), 1–14. doi: 10.1186/s12889-019-7969-5 32054474 PMC7020371

[9] Jeste D. V., Lee E. E., & Cacioppo S. (2020). Battling the modern behavioral epidemic of loneliness: suggestions for research and interventions. JAMA psychiatry, 77(6), 553–554. doi: 10.1001/jamapsychiatry.2020.0027 32129811 PMC7483387

[10] Hwang T. J., Rabheru K., Peisah C., Reichman W., & Ikeda M. (2020). Loneliness and social isolation during the COVID-19 pandemic. International psychogeriatrics, 32(10), 1217–1220. doi: 10.1017/S1041610220000988 32450943 PMC7306546

[11] What works Wellbeing. (2020). How has Covid-19 affected loneliness? Available from: https://whatworkswellbeing.org/resources/loneliness-lockdown-and-covid/ [Accessed 7th July 2021].

[12] Cacioppo J. T., Hawkley L. C., Crawford L. E., Ernst J. M., Burleson M. H., Kowalewski R. B., et al. (2002). Loneliness and health: Potential mechanisms. Psychosomatic medicine, 64(3), 407–417. doi: 10.1097/00006842-200205000-00005 12021415

[13] Fässberg M. M. et al. (2012). A systematic review of social factors and suicidal behavior in older adulthood. International Journal of Environmental Research and Public Health, 9, 722–745. doi: 10.3390/ijerph9030722 22690159 PMC3367273

[14] Kuiper J. S. et al. (2015). Social relationships and risk of dementia: A systematic review and meta-analysis of longitudinal cohort studies. Ageing Research Reviews, 22, 39–57. doi: 10.1016/j.arr.2015.04.006 25956016

[15] Steptoe, A., Shankar, A., Demakakos, P. and Wardle, J. (2013). Social isolation, loneliness, and all-cause mortality in older men and women. Proceedings of the National Academy Sciences of the United States of America, 110, 5797–5801. 10.1073/pnas.1219686110 PMC3625264 23530191

[16] Heffner K. L., Waring M. E., Roberts M. B., Eaton C. B. and Gramling R. (2011). Social isolation, C-reactive protein, and coronary heart disease mortality among community-dwelling adults. Social Science & Medicine, 72, 1482–1488. doi: 10.1016/j.socscimed.2011.03.016 21492978 PMC3090468

[17] Yu B., Steptoe A., Chen L.-J., Chen Y.-H., Lin C.-H. and Ku P.-W. (2020). Social isolation, loneliness, and all-cause mortality in patients with cardiovascular disease: a 10-year follow-up study. Psychosomatic Medicine, 82, 208–214. doi: 10.1097/PSY.0000000000000777 31842061

[18] Xia N. and Li H. (2018). Loneliness, Social Isolation, and Cardiovascular Health. Antioxidants & Redox Signaling, 28, 837–851. 28903579 10.1089/ars.2017.7312 PMC5831910

[19] Van den Bosch M., & Bird W. (2018). Oxford textbook of nature and public health: The role of nature in improving the health of a population. Oxford: Oxford University Press.

[20] Kobayashi L. C. and Steptoe A. (2018). Social isolation, loneliness, and health behaviors at older age: longitudinal cohort study. Annals of Behavioral Medicine, 52, 582–593. doi: 10.1093/abm/kax033 29860361 PMC6377432

[21] Polley M. J., & Pilkington K. (2017). A review of the evidence assessing impact of social prescribing on healthcare demand and cost implications. University of Westminster.

[22] Intelligent Health. (2021). Building Active Communities. Available from: http://www.intelligenthealth.co.uk/ [Accessed 22/07/2022].

[23] Harris M. A. (2018). The relationship between physical inactivity and mental wellbeing: Findings from a gamification-based community-wide physical activity intervention. Health psychology open, 5(1), 2055102917753853. doi: 10.1177/2055102917753853 29372067 PMC5774736

[24] Harris M. A. (2018). Beat the street: A pilot evaluation of a community-wide gamification-based physical activity intervention. Games for health journal, 7(3), 208–212. doi: 10.1089/g4h.2017.0179 29672165

[25] Harris M. A. (2019). Maintenance of behaviour change following a community-wide gamification based physical activity intervention. Preventive medicine reports, 13, 37–40. doi: 10.1016/j.pmedr.2018.11.009 30510892 PMC6260258

[26] Harris M. A., & Bird W. (2020). Bright spots, physical activity investments that work: Beat the Street. British journal of sports medicine, 54(8), 489–490. doi: 10.1136/bjsports-2018-099992 30323058

[27] Harris M. A., & Crone D. (2021). Using gamification to encourage active travel. Journal of Transport & Health, 23, 101275.

[28] Hughes S., Crone D., Sumner R., & Redmond M. (2019). Understanding well-being outcomes in primary care arts on referral interventions: a mixed method study. European Journal for Person Centred Healthcare, 7, 3.

[29] Creswell J.W., & Clark V.L.P. (2011). Designing and conducting mixed methods research. 2nd ed. Thousand Oaks, CA.: Sage Publications.

[30] Milton K., Cavill N., Chalkley A., Foster C., Gomersall S., Hagstromer M., et al. (2021). Eight investments that work for physical activity. Journal of Physical Activity and Health, 18(6), 625–630. doi: 10.1123/jpah.2021-0112 33984836

[31] Braun V., and Clarke V. (2006). Using thematic analysis in psychology. Qualitative research in psychology, 3(2), 77–101.

[32] Braun V., Clarke V., & Terry G. (2014). Thematic analysis. In Lyons A, & Rohleder P (Eds.), Qualitative Research in Clinical and Health Psychology. London: Palgrave MacMillan.

[33] Kaikati A.M., & Kaikati J.G. (2004). Stealth marketing: How to reach consumers surreptitiously. California Management Review, 46(4), 6–22.

[34] Evans W. D., & Hastings G. (Eds.). (2008). Public health branding: Applying marketing for social change. Oxford University Press.

[35] Summerbell C.D., Waters E., Edmunds L., Kelly S.A., Brown T., & Campbell K.J. (2005). Interventions for preventing obesity in children. Cochrane Database of Systematic Reviews, 3, CD001871. doi: 10.1002/14651858.CD001871.pub2 16034868

[36] Harris J.L., Pomeranz J.L., Lobstein T., & Brownell K.D. (2009). A crisis in the marketplace: how food marketing contributes to childhood obesity and what can be done. Annual Review of Public Health, 30, 211–25. doi: 10.1146/annurev.publhealth.031308.100304 18976142

[37] Manika D., Blokland Y., Smith L., Mansfield L., & Klonizakis M. (2021). Using stealth marketing techniques to increase physical activity and decrease sedentary time in the workplace: a feasibility study investigating the spill-overs of employee pro-environmental behaviour. International Journal of Business Science and Applied Management, 16(1), 28–49.

[38] Strömmer S., Shaw S., Jenner S., Vogel C., Lawrence W., Woods‐Townsend K., et al. (2021). How do we harness adolescent values in designing health behaviour change interventions? A qualitative study. British Journal of Health Psychology. [Online first]. doi: 10.1111/bjhp.12526 33945194

[39] Williamson C., Baker G., Mutrie N., Niven A., & Kelly P. (2020). Get the message? A scoping review of physical activity messaging. International Journal of Behavioral Nutrition and Physical Activity, 17, 1–15. doi: 10.1186/s12966-020-00954-3 32295613 PMC7160981

[40] Creswell J.W., & Clark V.L.P. (2011). Designing and conducting mixed methods research. 2nd ed. Thousand Oaks, CA.: Sage Publications.

[41] Slaney K. L., & Tafreshi D. (2018). Quantitative, qualitative, or mixed? Should philosophy guide method choice. Situating qualitative methods in psychological science, 27–42.

[42] Gelo O., Braakmann D., & Benetka G. (2008). Quantitative and qualitative research: Beyond the debate. Integrative psychological and behavioral science, 42(3), 266–290. doi: 10.1007/s12124-008-9078-3 18795385

[43] Lincoln Y. S., & Guba E. G. (1985). Naturalistic inquiry. Newbury Park, CA: Sage.

[44] Noblitt G. W., & Hare R. D. (1988). Meta-ethnography: Synthesizing qualitative studies. Newbury Park, CA: Sage.

[45] Rosenberg A. (1988). Philosophy of social science. Boulder, CO: Westview.

[46] Sandelowski M., Voils C. I., & Knafl G. (2009). On quantitizing. Journal of mixed methods research, 3(3), 208–222. doi: 10.1177/1558689809334210 19865603 PMC2768355

